# Pain assessment tools in paediatric palliative care: A systematic review of psychometric properties and recommendations for clinical practice

**DOI:** 10.1177/02692163211049309

**Published:** 2021-12-29

**Authors:** Adrienne YL Chan, Mengqin Ge, Emily Harrop, Margaret Johnson, Kate Oulton, Simon S Skene, Ian CK Wong, Liz Jamieson, Richard F Howard, Christina Liossi

**Affiliations:** 1 Centre for Safe Medication Practice and Research, Department of Pharmacology and Pharmacy, Li Ka Shing Faculty of Medicine, The University of Hong Kong, Pok Fu Lam, Hong Kong; 2 Laboratory of Data Discovery for Health (D24H), Hong Kong Science Park, Pak Shek Kok, Hong Kong; 3 Groningen Research Institute of Pharmacy, Unit of Pharmacotherapy, Epidemiology and Economics, University of Groningen, Groningen, The Netherlands; 4 Helen & Douglas House, Oxford, UK; 5 John Radcliffe Hospital, Oxford University Hospitals NHS Foundation Trust, Oxford, UK; 6 Department of Public Health and Primary Care, University of Cambridge, Cambridge, UK; 7 Great Ormond Street Hospital for Children NHS Foundation Trust, London, UK; 8 Surrey Clinical Trials Unit, University of Surrey, Surrey, UK; 9 Research Department of Pratice & Policy, University College London School of Pharmacy, London, UK; 10 Centre for Medicines Optimisation Research and Education, University College London School of Pharmacy and University College London Hospital, London, UK; 11 School of Psychology, University of Southampton, Southampton, UK

**Keywords:** Pain measurement, pain assessment, palliative care, paediatrics

## Abstract

**Background:**

Assessing pain in infants, children and young people with life-limiting conditions remains a challenge due to diverse patient conditions, types of pain and often a reduced ability or inability of patients to communicate verbally.

**Aim:**

To systematically identify pain assessment tools that are currently used in paediatric palliative care and examine their psychometric properties and feasibility and make recommendations for clinical practice.

**Design:**

A systematic literature review and evaluation of psychometric properties of pain assessment tools of original peer-reviewed research published from inception of data sources to April 2021.

**Data sources:**

PsycINFO via ProQuest, Web of Science Core, Medline via Ovid, EMBASE, BIOSIS and CINAHL were searched from inception to April 2021. Hand searches of reference lists of included studies and relevant reviews were performed.

**Results:**

From 1168 articles identified, 201 papers were selected for full-text assessment. Thirty-four articles met the eligibility criteria and we examined the psychometric properties of 22 pain assessment tools. Overall, the Faces Pain Scale-Revised (FPS-R) had high cross-cultural validity, construct validity (hypothesis testing) and responsiveness; while the Faces, Legs, Activity, Cry and Consolability (FLACC) scale and Paediatric Pain Profile (PPP) had high internal consistency, criterion validity, reliability and responsiveness. The number of studies per psychometric property of each pain assessment tool was limited and the methodological quality of included studies was low.

**Conclusion:**

Balancing aspects of feasibility and psychometric properties, the FPS-R is recommended for self-assessment, and the FLACC scale/FLACC Revised and PPP are the recommended observational tools in their respective age groups.

**What is already known about the topic?**

- Pain is a well-documented, highly prevalent symptom in infants, children and young people with life-limiting conditions.
- Assessing pain in infants, children and young people remains a challenge due to diverse patient conditions, types of pain and often a reduced ability or inability of patients to communicate verbally.
- No pain assessment tools are validated specifically for use within paediatric palliative care.

**What this paper adds?**

- This systematic review found no evidence of pain assessment tools that have been explicitly validated for the paediatric palliative care setting.
- The Faces Pain Scale-Revised (FPS-R) is recommended for self-assessment, and The Faces, Legs, Activity, Cry and Consolability scale (FLACC)/FLACC-Revised and Paediatric Pain Profile (PPP) are the recommended observational tools in their respective validated age groups.

**Implications for practice, theory or policy**

- A number of scales demonstrated high feasibility but were not recommended due to the lack of validation evidence in infants, children and young people with life-limiting conditions in paediatric palliative care settings.
- Validation data of pain assessment tools is a prerequisite to selecting an optimal tool to effectively assess pain in this population.
- Robust clinical validation of pain assessment tools in paediatric palliative care settings is urgently needed for the long-term improvement of pain management and quality of life in children at the end-of-life.

## Introduction

Connor et al.^1^ estimated a global prevalence of 21 million children who required paediatric palliative care. In England alone, there were approximately 86,625 infants, children and young people living with life-limiting conditions in 2017/18.^2,3^ Compared to adults, ‘infants, children and young people’ experience a wider range of life-limiting conditions with significantly greater prognostic uncertainties, unpredictable symptoms and variable timescales of disease progression. Hain et al.,^4^ using International Classification of Diseases-10 codes, compiled a directory of nearly 400 conditions that could limit the life of infants, children and young people according to the categorisation and definitions provided by the Association for Children’s Palliative Care/Royal College of Paediatrics and Child Health.^5^ Partly due to recent health technological advances, infants, children and young people with life-limiting conditions experience significantly longer disease trajectories and thus require paediatric palliative care over extended periods.

The ultimate goal of paediatric palliative care, unquestionably, is to enhance the quality of life for infants, children and young people and their families, with a cornerstone of holistic care being the prevention, early identification, comprehensive assessment and management of pain and other distressing symptoms.^6^ Pain is one of the most distressing and prevalent end-of-life symptoms experienced by patients.^7^ In paediatric palliative care settings of infants, children and young people with progressive malignant diseases, pain was experienced in over 70% to over 90% in the different populations from United States,^8^ Japan,^9^ Sweden^10^ and the United Kingdom.^11^ However, paediatric pain is particularly prone to under-diagnosis, and under- or suboptimal treatment.^12,13^ In the aforementioned American study,^8^ only 27% of all children who reported pain actually experienced pain relief. Despite practitioners’ experience and self-efficacy regarding pain assessment, barriers such as the fear of side effects, abuse and inappropriate use remain around effective analgesia management.^7,14,15^

Paediatric pain of any aetiology is a biopsychosocial phenomenon^16^ and the palliative pain experience in particular, or ‘total pain’, is a multidimensional experience that includes physical, psychological, social, spiritual and practical dimensions.^17^ Even though pain assessment is a critical first step for adequate pain management across treatment settings^18^ and is advocated by national^19^ and international guidelines,^20^ assessing pain in paediatric palliative care remains a challenge. This is due to diverse patient conditions, types of pain and often a reduced ability or inability of patients to communicate verbally. Furthermore, despite the vast amount of pain assessment tools available, there is an absence of tools explicitly for use in paediatric palliative care for infants, children and young people with life-limiting conditions. Therefore, a suitable tool for use with this group must either be developed specifically or adapted from polyvalent tools that have been validated in populations that include infants, children and young people with life-limiting conditions in paediatric palliative care. In addition, a range of validated tools needs to be available to meet the different developmental and communication needs of the paediatric palliative care population.

Numerous reviews have been conducted to summarise the development and clinical validation of paediatric pain assessment tools in various contexts.^21–24^ A recent systematic review of reviews by Andersen et al.^25^ identified 65 observational paediatric pain assessment tools. Birnie et al.^26^ evaluated 8 out of the 60 self-report pain intensity assessment tools identified. However, neither study focussed on paediatric palliative care settings. Paediatric palliative care settings may include a variety of care settings, such as any tertiary care facilities, emergency rooms, community health centres, hospice facilities or even the children’s home that offer support to ‘*the care of children and families facing chronic life limiting illnesses*’.^27^ Another review conducted by Batalha et al.^28^ identified 17 pain assessment tools that assessed pain in children with cancer but the review did not produce any conclusive recommendations regarding the optimal pain assessment tools for use in this population. Furthermore, the study excluded paediatric patients with cognitive impairment, limiting the generalisability of the findings to a paediatric palliative care population.

The aims of this systematic review were to evaluate the psychometric properties of current age-specific pain assessment tools validated in various populations of children with life-limiting and life-threatening illnesses receiving palliative care and make recommendations for clinical practice. **More specifically we addressed the following review questions:**

- What pain assessment tools have been used to assess pain in populations of children with life-limiting and life-threatening illnesses receiving palliative care?
- What are the psychometric properties of these tools? This includes the validity, reliability and responsiveness of the pain assessment tools according to the COnsensus-based Standards for the selection of health Measurement Instruments (COSMIN).
- What are the most suitable pain assessment tools for use in paediatric palliative care?

## Methods

We undertook a systematic review and narrative synthesis of peer-reviewed studies published in full and in English since the inception of the respective electronic databases, PsycINFO via ProQuest, Web of Science Core, Medline via Ovid, EMBASE, BIOSIS and CINAHL, up to April 2021. The reference lists of included journal articles, and existing reviews were hand searched. The review was reported in accordance with the Preferred Reporting Items for Systematic reviews and Meta-Analyses (PRISMA) and the COnsensus-based Standards for the selection of health Measurement INstruments (COSMIN) guidelines.^29,30^

### Search strategy

The search strategy was developed based on suggestions from the COSMIN guidelines, incorporating construct, population and instrument search together with the COSMIN psychometric properties filter (see Supplemental File 1).^30–32^ Combinations of keywords, text words, Medical Subject Headings (MeSH) and other terms relevant to the four components were chosen for each database to optimise the sensitivity and specificity of the search. The thesaurus vocabulary of each database was used to adapt the search terms. The search terms were then combined with the COSMIN search filters (available on http://www.cosmin.nl/).^32^ Also incorporated in the search strategy, were search filters such as that developed by the National Institute for Health and Care Excellence (NICE), the Palliative Care Search Filter (PCSF) and relevant systematic reviews conducted by Beecham et al. (2016) and Anderson et al. (2017).^25,33–35^

### Inclusion/exclusion criteria

Study eligibility criteria were developed according to the COSMIN guidelines^30^ (see Table 1). All published peer-reviewed studies, regardless of study design, that reported the use of pain assessment tools in paediatric palliative settings completed by the parent or clinician of the infants, children and young people or the patient themselves were considered for inclusion in the review. Studies were excluded if they were not published in English, did not describe scientific research, were not peer-reviewed articles, or if they were not conducted in end-of-life care, palliative care or hospice care settings. We also excluded studies that used an assessment tool that could not be replicated due to the lack of clarity in methodology, version used or statistics.

**Table 1.** Eligibility criteria according to the COSMIN guidelines^30^ for the systematic review of pain assessment tools used in paediatric palliative care.

Criteria	Inclusion criteria	Exclusion criteria
Construct	Pain	The tool does not assess pain
Population	Infants, children and young people aged 0–18 with life-limiting conditions	The study sample (or an arbitrary majority, e.g. ⩾50%) does not represent infants, children and young people aged 0–18 with life-limiting conditions
Instrument	Pain assessment tools	Not applicable (all assessment tools are considered)
Psychometric properties	COSMIN defined Validity, Reliability, Responsiveness, Interpretability, Acceptability Measures	The study does not aim to evaluate one or more psychometric properties of a pain assessment tool, its development or its interpretability

### Extraction

Two researchers (AC and MG) independently assessed study eligibility and undertook data extractions simultaneously. A standardised data extraction form modified from the COSMIN guidelines^30^ was used to record information on the context, population and outcomes and psychometric properties of each study. The COSMIN taxonomy, terminology and definitions of measurement properties for health-related patient-reported outcomes was used to appraise the psychometric properties of the instruments reviewed.^36^ COSMIN provides a consensus on terminology surrounding psychometric properties and a checklist for evaluating the methodological quality of studies reporting on validity, reliability and responsiveness.^37^ To synthesise the results from the included studies, the following data were extracted from each paper and organised into tables: authors, year and country of publication, journal, aim, sample size and characteristics, study setting, study design, measure(s), outcomes and psychometric properties.

### Risk of bias and quality of the results assessment of individual studies

The methodological quality of each included study was assessed according to the COSMIN Risk of Bias Checklist.^38^ For all studies, each psychometric property was rated against the standards of quality listed in boxes 3–10 of the checklist to screen for methodological flaws that could lead to bias. Each question in the respective boxes was given the answer ‘Very good’, ‘Adequate’, ‘Doubtful’, ‘Inadequate’ or ‘Not applicable’, on the scoring form provided on the COSMIN website.^32^ Combining the individual answers on 98 items (5–18 items per psychometric property), each psychometric property per study was given a rating of ‘Very good’, ‘Adequate’, ‘Doubtful’ or ‘Inadequate’. As there is no consensus on the gold standard for pain assessment tools in paediatric palliative care settings currently, all self-report measures are considered the gold standard when assessing criterion validity, which assesses the degree to which the measures are an adequate reflection of a ‘gold standard’.

Each psychometric property per study was rated against the latest criteria for good psychometric properties on the COSMIN guidelines (Table 2)^30^ and was then given a rating of ‘sufficient’ (+), ‘insufficient’ (−) or ‘indeterminate’ (?).

**Table 2.** Psychometric properties recorded according to COSMIN guidelines.

Domain	Psychometric property	Aspect of a psychometric property	Definition
Reliability			The degree to which the measurement is free from measurement error
Reliability (extended definition)			The extent to which scores for patients who have not changed are the same for repeated measurement under several conditions: for example using different sets of items from the same PROM (internal consistency); over time (test-retest); by different persons on the same occasion (inter-rater); or by the same persons (i.e. raters or responders) on different occasions (intra-rater)
	Internal consistency		The degree of the interrelatedness among the items
	Reliability		The proportion of the total variance in the measurements which is due to ‘true’ differences between patients
	Measurement error		The systematic and random error of a patient’s score that is not attributed to true changes in the construct to be measured
Validity			The degree to which a PROM measures the construct(s) it purports to measure
	Content validity		The degree to which the content of a PROM is an adequate reflection of the construct to be measured
		Face validity	The degree to which (the items of) a PROM indeed looks as though they are an adequate reflection of the construct to be measured
	Construct validity		The degree to which the scores of a PROM are consistent with hypotheses (for instance with regard to internal relationships, relationships to scores of other instruments or differences between relevant groups) based on the assumption that the PROM validly measures the construct to be measured
		Structural validity	The degree to which the scores of a PROM are an adequate reflection of the dimensionality of the construct to be measured
		Hypotheses testing	Item construct validity
		Cross-cultural validity	The degree to which the performance of the items on a translated or culturally adapted PROM are an adequate reflection of the performance of the items of the original version of the PROM
	Criterion validity		The degree to which the scores of a PROM are an adequate reflection of a ‘gold standard’
Responsiveness			The ability of a PROM to detect change over time in the construct to be measured
	Responsiveness		Item responsiveness
Interpretability*			Interpretability is the degree to which one can assign qualitative meaning – that is, clinical or commonly understood connotations – to a PROM’s quantitative scores or change in scores.
			

* Although an important property of an instrument, interpretability is not a psychometric property.

### Data synthesis and risk of bias across studies

Due to the heterogeneity of the studies, the results were qualitatively summarised together with considerations of the quantitative significance of each finding. Quantitative pooling of results was not conducted. The summarised evidence of psychometric properties per tool was rated against the same criteria for good psychometric properties with regards to the strength of evidence. The risks of bias across studies were graded using the Grades of Recommendation, Assessment, Development and Evaluation (GRADE) approach, where summarised evidence was downgraded based on five factors of insufficient quality of evidence. These were: risk of bias, inconsistency of results of studies, indirectness of populations validated compared to our population of interest and imprecision of results.^38,39^ Initial ratings from AC and GG had fair interrater agreement (Kappa = 0.39 for psychometric property results and 0.30 for GRADE ratings). Discrepancies in results were further discussed in a consensus meeting (between AC and GG) where absolute agreement was reached.

The final recommendations for clinical practice and research were discussed and agreed by both clinicians and researchers, co-authors AC, EJ, CL, RH, EH, IW who are members of the DIPPER study. The DIPPER study is a four-phase feasibility study of a randomised clinical trial of transmucosal diamorphine versus oral morphine for breakthrough pain in children and young people with life-limiting conditions.

## Results

**Figure 1.**
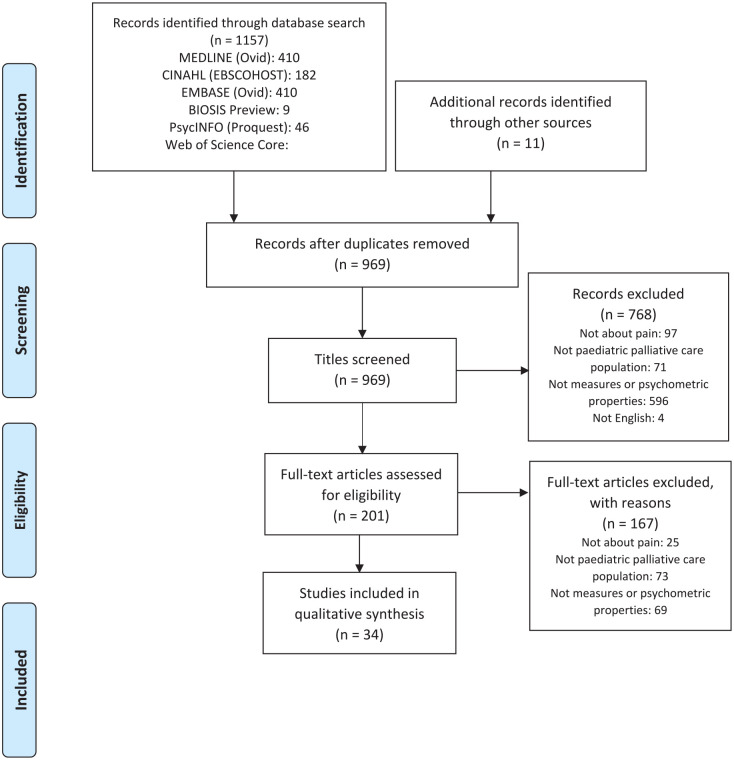
PRISMA flow diagram of records identified in the systematic review of pain assessment tools used in paediatric palliative care.

Figure 1 presents the flow of studies through the review. The initial search yielded 1157 articles with a further 11 articles from the manual search of reference lists of included studies. A total of 1168 articles were assessed for eligibility. After removing 199 duplicates, 969 titles and abstracts were screened against the eligibility criteria. Two hundred and one full-text articles were retrieved and reviewed independently by two researchers (AC and GG).

One hundred and sixty-seven articles were further excluded either because they did not describe a tool that measured pain (*n* = 25), the study sample did not represent a paediatric palliative care population (*n* = 73) or the study did not evaluate any psychometric properties (*n* = 69). A total of 34 articles were included in the review.

### Pain measurement tools and study populations

A total of 22 pain assessment tools were evaluated in this review. Symptoms Screening in Paediatrics (SSPedi) had both self-report and proxy report versions. Characteristics of all included measures and populations are listed in Table 3 for observational and self-report tools. Apart from the Oral Mucositis Daily Questionnaire (OMDQ),^40^ none of the measures included were disease-specific. Mirroring the distribution of life-limiting conditions in paediatric palliative care, the majority of the tools were developed, or validated, in children with cancer. Other populations included children with haematological, neurological and surgical conditions, and those requiring intensive care.

**Table 3.** Characteristics of included measures.

Self-report scales														
Measure, country of origin (reference to first article)	Acronym	Studies included	Construct(s) primary (secondary)	Age range in years unless specified	Target population	Recall period	(Sub)scale (s) (number of items)	Completion time	Training time	Response options	Range of scores/scoring	Original language	Available translations	
Adolescent paediatric Pain Tool, U.S. (Savedra et al. )^41^	APPT	Fernandes et al.^42^; Özalp Gerçeker et al., 2018^43^; Madi and Badr,^44^	Pain – postoperative	I: 8–17 S: 7–18^43^ 8–17^42,44^	Surgery, Post-operative pain	Current or most recent pain	3 (3)	3.2–6.4 minutes	n.r.	43 body segments; 5 words- Word Graphic Rating Scale; 56–67 pain descriptors	(1) Any location on the body outline; (2) no pain to worst possible pain; (3) 0–67 pain descriptors	English	Spanish, Portuguese, Turkish, Arabic	
Children’s Procedural Interview, U.S. (Pfefferbaum et al. )^45^	CPI	Pfefferbaum et al.^45^	Pain – procedural, anxiety	I: 3–15 S: 9–15.9	Cancer; Procedural pain	<30 minutes	n.r.	n.r.	n.r.	n.r.	Semi-structured with open answers	English/ Spanish	n.r.	
The DOLLS tool, Lebanon (Badr Zahr et al. )^46^	DOLLS	Zahr et al.^46^	Pain – procedural	I: 4–10 S: <1 (11 months), 4–10	Cancer; Procedural pain	0	1 (1)	n.r.	n.r.	6 fabric dolls	6 ordinal dolls	Arabic	n.r.	
Faces scales, U.S. (LeBaron and Zeltzer,)^47^	FACES	LeBaron and Zeltzer^47^; Pfefferbaum et al.^45^	Pain – acute, anxiety	I: 6–18 S: 2–6	Cancer; Procedural pain (BMA, LP)	0	1 (1)	n.r.	1 month	5 faces (image)	1–5	English	n.r.	
Faces Pain Scale – Revised, Canada (Hicks et al. )^48^ Faces Pain Scale (Bieri et al. )^49^	FPS-R	Hicks et al.^48^; Miro and Huguet^50^; da Silva et al.^51^	Pain	4–12	Unspecified diseases	n.r.	1 (1)	n.r.	n.r.	6 faces (image)	0–10	English	Arabic, Bulgarian, Chinese, Dutch, French, German, Hebrew, Italian, Persian (Farsi), Portuguese, Spanish, Swedish and Thai	
Memorial Symptom Assessment Scale for children aged 7–12 years, Australia (Collins et al. )^52^	MSAS 7–12	Collins et al.^52^	Symptoms	7–12	Cancer	n.r.	1 (8)	5.8 minutes	0	Yes/No, 3-point scales, 4-point scales	Yes/No, 1–3, 0–3	English	n.r.	
Poker Chip Tool/Pieces of Hurt U.S. (Hester et al. )^53^	PCT	West et al.^54^	Pain – procedural	I: 4.6–6.7	Healthy, PICU	0	1 (1)	n.r.	n.r.	4 poker chips	0–4	English	n.r.	
Pain Squad, Canada (Stinson)^55^	n.r.	Stinson^55^; Stinson et al.^56^	Pain – unspecified	9–18	Cancer	n.r.	1 (20)	n.r..	n.r.	Visual analogue slider scale, selectable body-map, multiple-choice question, a free-text question.	1) 0–10 2) pain locations (e.g. left, right) 3) multiple choices 4) open answer	English	n.r.	
Pain Interference Index, Sweden (Wicksell et al. )^57^	PII	Martin et al.^58^	Pain interference – longstanding pain	I: 10–18 S: 6–25	Pain syndrome	2 weeks	1 (6)	1–2 minutes	n.r.	7–point scale	0–6	Swedish	English	
Rainbow Pain Scale, Canada (Mahon et al. )^59^	RPS	Mahon et al.^59^	Ongoing Pain	5–10	Cancer or haematological diseases	n.r.	1 (5)	n.r.	n.r.	24 colours	A box of 24 standard colours provided by Crayola	English	n.r.	
Symptom Screening in paediatrics Tool (self-report), Canada (Dupuis et al. )^60^	SSPedi	Dupuis et al.^60^	Symptoms	8–18	Cancer or hematopoietic stem aaaaaaaaa transplant (HSCT) recipients	n.r.	1 (15)	n.r.	n.r.	5–point scale	0 (no bother)–4 (worst bother)	English	n.r.	
Wong-Baker FACES pain rating scale, U.S. (Wong and Baker)^61^	WBS	Wong and Baker^61^; West et al.^54^; Holdsworth et al.^62^; Wiener et al.^63^	Pain – unspecified	I: 3–18 S: 5–13 ^54^ 7–21 ^63^	Hospitalised children	n.r.	1 (1)	n.r.	n.r.	6 one-dimensional faces	0–5	English	n.r.	
Oral Mucositis Daily Questionnaire, U.S. (Stiff et al. )^64^	OMDQ	Tomlinson et al.^65^; Manji et al.^40^	Pain and daily functioning	I: >18 S: 1–11	Cancer	0	3 (10)	n.r..	n.r.	5-point scale, 11-point scale	0–4, 0–10	English	n.r.	
Observational scales														
Measure, country of origin (reference to first article)	Acronym	Studies included	Construct(s) Primary (Secondary)	Age range in years unless specified	Target population	Mode of administration i.e. reported by parent/ nurse/ doctor/ researcher	Recall period	(Sub)scale (s) (number of items)	Completion time	Training time	Response options	Range of scores /scoring	Original language	Available translations
COMFORT Scale, U.S. (Ambuel et al. )^66^	COMFORT	Ambuel et al.^66^; van Dijk et al., 2000^67^; Van Dijk et al.^68^	Distress (Pain – postoperative)	I: n.r. S: 0–3 (van Dijk et al., 2000); 0–3^68^	PICU, postoperative pain	Parent	0–2 minutes	8 (40)	2 minutes	2 hours	5-point scale	1–5	English	n.r.
Douleur Enfant Gustave Roussy Scale/The Gustave Roussy Child Pain Scale and revised version, France (Gauvain-Piquard et al. )^69^	DEGR	Gauvain-Piquard et al.^69^; Gauvain-Piquard et al.^70^; Marec-Berard et al.^71^	Pain – acute (depression, anxiety)	I: 2–6 S: 2–6	Cancer	Nurse or researcher	n.r.	3 (17)	5–10 min	2–3 hrs	5-point scales, with 5 respective descriptions of increasing severity with reference to a provided definition of the item.	0–4	French	n.r.
The Faces, Legs, Activity, Cry and Consolability pain assessment tool, U.S. (Merkel et al. )^72^	FLACC	Merkel et al.^72^; Manworren and Hynan^73^; Da Silva et al.^51^	Pain	n.r.	Surgery	Researcher	n.r.	1(5)	5 minutes	n.r.	3-point scale	0–2	English	Portuguese
Hétéro-Évaluation de la Douleur de l’Enfant, France (Marec-Berard et al. )^71^	HEDEN	Marec-Berard et al.^71^	Pain – prolonged	2–6	Cancer	Nurse	n.r.	3 (6)	4.42 (5.9) min	n.r.	3-point scale	0–2	French	n.r.
Modified Infant Pain Scale, U.S. (Buchholz et al. )^74^	MIPS	Buchholz et al.^74^	Pain – postoperative	4–30 weeks	Surgery	Nurse	0	1(13)	n.r.	2 hours	3-point scale	0–2	English	n.r.
Objective Pain Scale, U.S., (West et al. )^54^	OPS	West et al.^54^	Pain	5–13	Cancer	Parent and nurse	0	1 (5)	n.r.	n.r.	3-point scale	0–2	English	n.r.
Procedure Behaviour Check List, U.S., (LeBaron and Zeltzer)^47^	PBCL	LeBaron and Zeltzer)^47^; Pfefferbaum et al.^45^	Pain – acute, anxiety	6–17	Cancer; Procedural pain (BMA, LP)	Unspecified	0	1 (8) repeated for three time periods	Time 1: 4–6 minutes, Time 2: 2–3 minutes, Time 3: 2–4 minutes	1 month	5-point scales, with provided definitions of each behavioural category	1–5	English	n.r.
Pain Interference Index- Parent, U.S. (Martin et al. )^58^	PII-P	Martin et al.^58^	Pain interference – chronic pain	6–25	Neurofibromatosis type 1 or Cancer	Parent	n.r.	1 (6)	1–2 minutes	n.r.	7-point scale	0–6	English	n.r.
Paediatric Pain Profile, U.K. (Hunt et al. )^75^	PPP	Hunt et al.^75^; Hunt et al.^76^; Pasin et al.^77^	Pain	1–18	Neurological and cognitive impairments, unable to communicate through speech or any augmentative device	Parent	Retrospective	1 (20)	n.r.	n.r.	4-point ordinal scale	0–3	English	Portuguese
Symptom Screening in paediatrics Tool (proxy-report), Canada (Dupuis et al. )^60^	SSPedi	Dupuis et al.,^60^ Hyslop et al.^78^	Symptoms	8–18	Cancer or hematopoietic stem cell transplant (HSCT) recipients	n.r.	1 (15)	n.r.	n.r.	5-point scale	0 (no bother)–4 (worst bother)	English	n.r.	English

I: intended population where tool was first published; S: populations reported in subsequent studies where tool was validated; PICU: paediatric intensive care unit; n.r.- not reported; APPT: Adolescent Paediatric Pain Tool; CPI: Children’s Procedural Interview; DEGR scale: Douleur Enfant Gustave Roussy; FPS-R: Faces Pain Scale-Revised; FLACC scale: Face, Legs, Activity, Cry, Consolability scale; HEDEN scale: Hétero Evaluation Douleur Enfant scale; MIPS: Modified Infant Pain Scale; MSAS: Memorial Symptom Assessment Scale; OMDQ: Oral Mucositis Daily Questionnaire; OPS: Objective Pain Scale; PBCL: Pain Behaviour Check List; PCT: Poker Chip Tool; PII: Pain Interference Index; PII-P: Pain Interference Index-Parent; PPP: Paediatric Pain Profile; *r*: Pearson product moment correlation coefficient; RPS: Rainbow Pain Scale; SSPedi: Symptom Screening in Paediatrics; WBS: Wong-Baker FACES Pain Rating Scale; U.K.: United Kingdom; U.S. : United States; NF-1: neurofibromatosis type 1.

### Feasibility of use

Feasibility of use of tools was assessed by evaluating their respective types and ease of administration, completion time, training time, recall time, length of the instrument, ease of scoring, calculation and standardisation. Given the diversity and needs in patients with life-limiting conditions, there is no single feasible tool.

Completion time was reported in all of the self-report tools apart from the paediatric pain profile (PPP), and ranged from 1 to 10 min. Completion time was not reported for most observational tools. Training time and recall period were generally not very well reported. Most tools adopt point scales or other forms of ordinal responses, with the adolescent paediatric pain tool (APPT), children’s procedural interview (CPI) and Pain Squad incorporating semi- structured or open answers in the response options. All tools that used an ordinal response system were relatively easier to standardise, calculate and administer. Of all reported tools, the Pain Squad and the Symptoms Screening in Paediatrics (SSPedi) were developed as electronic versions to be administered using mobile devices.

### Methodological quality

Ratings for methodological quality of psychometric properties per study are illustrated in Supplemental File 2. Internal structure was assessed by the reporting of content validity, structural validity, cross-cultural validity and internal consistency. Internal consistency was the property most frequently reported to demonstrate internal structure of each tool. Most studies also described hypothesis testing properties, convergent and divergent validity. Although reliability measures were often reported, when referencing dichotomous, nominal or ordinal scores, a number of studies failed to demonstrate evidence of kappa calculations and weighting schemes,^43^ while some only reported the Pearson or Spearman correlation coefficient without any kappa calculation.^40,65,71^ This significantly discounted the strength of reported evidence. Only one study referred to intrarater agreement.^76^

Most of the included studies provided limited evidence of psychometric properties in their respective populations, none reported information on all four domains or more than half of the psychometric properties of interest. Psychometric property results and respective methodological quality ratings of individual studies are summarised in Supplemental File 2. A summary of the qualitatively pooled findings can be found in Table 4.

**Table 4.** Overall ratings of qualitatively summarised psychometric properties and quality of the evidence per pain measure.

Measure	Content validity	Structural validity	Cross-cultural validity	Internal consistency	Hypotheses testing	Criterion validity	Reliability	Measurement error	Responsiveness
APPT	? (Low)	NA	? (Low)	+ (Moderate)	NA	NA	+ (High)	NA	NA
COMFORT	+ (Moderate)	+ (Moderate)	NA	+ (High)	+ (Low)	NA	+ (High)	NA	+ (Low)
CPI	NA	NA	NA	+ (Moderate)	− (Moderate)	NA	NA	NA	− (Moderate)
DEGR	NA	+ (High)	NA	+ (High)	− (Moderate)	NA	+ (Moderate)	NA	− (Moderate)
DOLLS	NA	NA	NA	NA	+ (Moderate)	+ (High)	NA	NA	+ (Moderate)
FLACC	NA	NA	NA	+ (Moderate)	+ (Moderate)	+ (Low)	+ (High)	NA	+ (Moderate)
Le Baron and Zeltzer Faces Scale	NA	NA	NA	+ (High)	? (Very low)	NA	NA	NA	? (Very low)
FPS-R	NA	NA	+ (Very low)	NA	+ (Moderate)	− (Moderate)	NA	NA	+ (Very low)
HEDEN	NA	NA	NA	− (Moderate)	− (Moderate)	NA	− (Very low)	NA	− (Moderate)
MIPS	NA	NA	NA	NA	NA	+ (Very low)	− (Very low)	NA	NA
MSAS (7–12)	NA	NA	NA	− (Moderate)	NA	+ (Moderate)	− (Moderate)	NA	NA
OMDQ	NA	NA	NA	NA	+ (High)	NA	+ (Low)	NA	+ (High)
OPS	NA	NA	NA	NA	NA	NA	NA	NA	− (Very low)
Pain Squad	+ (Low)	NA	NA	+ (High)	− (High)	NA	NA	NA	− (Moderate)
PBCL	NA	NA	NA	+ (Very low)	− (Moderate)	− (Moderate)	? (Low)	NA	− (Low)
PCT	NA	NA	NA	NA	+ (Very low)	− (Low)	? (Very low)	NA	− (Low)
PII	NA	NA	NA	+ (Moderate)	− (Moderate)	NA	NA	NA	− (Moderate)
PII (PII-P)	NA	NA	NA	+ (Moderate)	NA	− (Moderate)	NA	NA	NA
PPP	+ (Very low)	NA	+ (High)	+ (High)	− (Moderate)	+ (Moderate)	+ (Moderate)	NA	+ (Low)
RPS	NA	NA	NA	NA	NA	+ (Moderate)	+ (Low)	NA	+ (Low)
SSPedi	NA	NA	NA	+ (Moderate)	− (Low)	NA	+ (Moderate)	NA	− (Low)
WBS	NA	NA	NA	NA	+ (High)	+ (High)	? (Low)	NA	+ (High)

+ : sufficient overall rating psychometric properties; ?: indeterminate overall rating psychometric property; −: insufficient overall rating psychometric property; NA: information not available; APPT: Adolescent Paediatric Pain Tool; CPI: Children’s Procedural Interview; DEGR scale: Douleur Enfant Gustave Roussy; FPS-R: Faces Pain Scale-Revised; FLACC scale: Face, Legs, Activity, Cry, Consolability scale; HEDEN scale: Hétero Evaluation Douleur Enfant scale; MIPS: Modified Infant Pain Scale; MSAS: Memorial Symptom Assessment Scale; NA: not available; OMDQ: Oral Mucositis Daily Questionnaire; OPS: Objective Pain Scale; PBCL: Pain Behaviour Check List; PCT: Poker Chip Tool; PII: Pain Interference Index; PII-P: Pain Interference Index-Parent; PPP: Paediatric Pain Profile; *r*: Pearson product moment correlation coefficient; RPS: Rainbow Pain Scale; rs: Spearman correlation coefficient; SSPedi: Symptom Screening in Paediatrics; τ: Kendall’s tau (τ) correlation coefficient; WBS: Wong-Baker FACES Pain Rating Scale.

Content validity was assessed in five of the studies for four of the tools and generally there was appropriate evaluation on relevance and comprehensibility in patients and experts. Structural validity was assessed in four of the studies for two of the tools and generally there was appropriate use of confirmatory or exploratory factor analysis except for one study which used Principal Component Analysis.^66^ Cross cultural validity was assessed in four of the studies for three of the tools utilising either independent translation, back-translation or expert committee. Internal consistency was assessed in 14 of the studies for 16 of the tools and generally there was appropriate use of Cronbach’s alpha. Hypothesis testing was assessed in all of the tools except RPS^59^ and generally there was appropriate use of convergent and divergent validity. Criterion validity was assessed in 14 of the studies for 14 of the tools and generally there were assessments on either concurrent or predictive validity. Reliability was assessed in 19 of the studies for 14 of the tools and in general there was appropriate use of test-retest reliability, interrater agreement and intrarater agreement.

## Pain assessment tools recommended for clinical practice

### Self-report

#### The faces pain scale-revised

The Faces Pain Scale-Revised (FPS-R) consists of six drawings of faces, arranged in a horizontal row, with a neutral face at the left (score of 0) and the maximum pain face at the right (score of 10). The tool was validated in a clinical sample of 5- to 17-year-olds who were admitted to the hospital with cancer. Brazilian and Catalan versions of the tool were examined. The tool demonstrated strong evidence of criterion and construct validity. The internal structure of the tool was not well examined. The FPS-R cross-cultural validity was demonstrated with the development process of the Catalan version. However, the tool was not validated culturally after the translation process.

### Observational tools

#### The faces, legs, activity, cry and consolability scale (FLACC)

The FLACC scale measures pain intensity by rating five behaviours (face, legs, activity, consolability and cry) each scored from 0 to 2 to derive a total score from 0 to 10. The descriptors for each item are considered indicative of behaviours exhibited by children in pain and the descriptors associated with each score level (0, 1 or 2) seen to represent an escalation consistent with increasing pain intensity. The tool was initially developed to measure distress and widely adopted for measuring post-operative pain. It was validated in 4- to 17-year-olds. English, Arabic and Brazilian Portuguese versions are examined in this review. The tool has good internal consistency and reliability. The Brazilian Portuguese version of the FLACC demonstrated sufficient internal consistency in 7- to 17-year-olds; other measures of internal structure were not reported. The original English version possesses high interrater agreement.

#### Paediatric pain profile

The Paediatric Pain Profile (PPP) is a 20-item rating scale. Each item is rated on a four-point scale as occurring ‘not at all’ to ‘a great deal’ in any given time period. The total score ranges from 0 to 60. The tool is validated in 1- to 18-year-olds with neurological and cognitive impairment. In the Brazilian version of the PPP, content validation was conducted with health professionals and primary caregivers. The consensus yielded a clarity rating between clear and very clear. The tool has very good criterion validity when using the numerical rating scale as a gold standard. Reliability was assessed in terms of interrater agreement, interrater agreement and test-retest reliability in various studies. Overall, the tool has good reliability, good internal consistency and insufficient convergent validity with physiological measures.

## Discussion

### Main findings

This is the first systematic review of pain assessment tools currently being used in paediatric palliative care, together with an examination of their psychometric properties and feasibility allowing us to make recommendations for clinical practice. Balancing aspects of feasibility and psychometric properties, the FPS-R is the recommended self-report measure while PPP and FLACC are the recommended observational tools. The revised FLACC R^79^ scale is an observational pain tool based on the FLACC scale modified to include additional pain behaviours often found in children who are non-verbal or with cognitive impairment. It includes additional items reported by parents related to their child’s individualised behaviours within each category. Although no studies in this review have included FLACC R, we recommend it for children who are non-verbal or with cognitive impairment.

As pain is a subjective experience, whenever possible, self-report from infants, children and young people should be the first-choice method of pain assessment.^18^ Infants, children and young people with life-limiting conditions are often cared for in non-clinical settings, therefore parent or carer reports should be regarded as the next choice of pain assessment. Our recommendations prioritise tools that have been validated with a high correlation between parent and self-report. We recommend all pain assessment tools that demonstrated sufficient content validity and internal consistency in infants, children and young people with life-limiting conditions population where results truly reflect patients’ pain intensity. As indicated by the COSMIN guidelines, measures with high quality evidence for insufficient psychometric properties are not recommended for use in the defined target population, infants, children and young people with life-limiting conditions, until a modified version of the tool is proven valid and reliable in that population.

### Strengths and limitations

This is the first systematic review that has followed an established methodology to summarise and critically appraise the extant literature pertaining to the reliability, validity, responsiveness and feasibility of pain assessment tools in paediatric palliative care. Clinicians routinely need to make complex decisions and identify the appropriate pain measurement tool for different patient populations. Systematic reviews of psychometric properties provide the necessary evidence base to underpin these decisions.

### What this study adds

In their review, Coombes et al.,^80^ reported a range of challenges associated with the inherent limitations of the COSMIN checklist, including but not limited to, the content validation process of the checklist, its interrater reliability and the ambiguity of inadequate reporting and inadequate quality of each psychometric property.

This review examines the feasibility of pain measures by looking at each tool’s intrinsic characteristics (Table 3) such as recall period, completion time, number of items, mode of administration, settings in which the tool has been validated and other factors. Due to insufficient information available from the existing literature, other extrinsic relevant aspects were not thoroughly examined. These include: patient’s and clinicians’ comprehensibility, copyright and regulatory agency’s requirement for approval. Interpretability was not examined in pain tools due to the fact that most tools aimed to measure one dimension of pain that is, pain intensity.

By default, pain measurement tools are developed to help standardise reporting and inform clinicians and carers of pain management decisions in a way that is generalisable and comprehensible to a third party who is not experiencing the pain. The pain tools recommended in this review were chosen as a result of weighing up the feasibility and psychometric properties of the tools. As such, the tools may not be optimal in the measurement of all types of pain experienced by infants, children and young people in palliative care such as breakthrough pain^81^ for example.

*Clinical implications and future directions* Despite the vast amount of pain assessment tools developed and the numerous studies on the utilisation of these tools, our search found few that focussed on the validation of these tools in infants, children and young people with life-limiting conditions in paediatric palliative care settings. A number of scales demonstrated high levels of feasibility but were not recommended due to the lack of validation evidence in infants, children and young people with life-limiting conditions or paediatric palliative care settings. Validation data of pain assessment tools is a prerequisite to the selection of an optimal tool to effectively assess pain in this population. Given the significant distress caused by pain, and its effect on the quality of life of children particularly at the end-of-life, implementation and standardisation of pain assessment is urgently needed. This calls for robust clinical validation efforts in paediatric palliative care settings for the long-term improvement of pain management.

## Supplemental Material

sj-pdf-1-pmj-10.1177_02692163211049309 – Supplemental material for Pain assessment tools in paediatric palliative care: A systematic review of psychometric properties and recommendations for clinical practice Click here for additional data file. Supplemental material, sj-pdf-1-pmj-10.1177_02692163211049309 for Pain assessment tools in paediatric palliative care: A systematic review of psychometric properties and recommendations for clinical practice by Adrienne YL Chan, Mengqin Ge, Emily Harrop, Margaret Johnson, Kate Oulton, Simon S Skene, Ian CK Wong, Liz Jamieson, Richard F Howard and Christina Liossi in Palliative Medicine

sj-pdf-2-pmj-10.1177_02692163211049309 – Supplemental material for Pain assessment tools in paediatric palliative care: A systematic review of psychometric properties and recommendations for clinical practice Click here for additional data file. Supplemental material, sj-pdf-2-pmj-10.1177_02692163211049309 for Pain assessment tools in paediatric palliative care: A systematic review of psychometric properties and recommendations for clinical practice by Adrienne YL Chan, Mengqin Ge, Emily Harrop, Margaret Johnson, Kate Oulton, Simon S Skene, Ian CK Wong, Liz Jamieson, Richard F Howard and Christina Liossi in Palliative Medicine
