# Supplementary material for: Pain assessment tools in paediatric palliative care: A systematic review of psychometric properties and recommendations for clinical practice
Source: Palliat Med. 2021 Dec 29;36(1):30–43. doi: 10.1177/02692163211049309 (PMC8796159; doi:10.1177/02692163211049309)
Supplement: sj-pdf-1-pmj-10.1177_02692163211049309 – Supplemental material for Pain assessment tools in paediatric palliative care: A systematic review of psychometric properties and recommendations for clinical practice [file sj-pdf-1-pmj-10.1177_02692163211049309.pdf]

Supplementary File 1. Search Strategy used for electronic databases for systematic review of psychometric properties of pain assessment tools in paediatric palliative care (Searched September 2019)

Medline via Ovid
1. exp Pain/
2. Pain Management/
3. (pain* or headache* or migraine* or neuralgia or neuropathic or myalgia or metatarsalgia or sciatica).ab,ti.

4. or/1-3
5. exp Infant/
6. exp Child/
7. exp PEDIATRICS/ or exp PUBERTY/
8. ADOLESCENT/ or MINORS/
9. (neonate* or newborn or infant* or child* or adolescen* or paediatric* or pediatric* or baby or babies or toddler* or teen* or youth$ or young or highschool$ or juvenile* or boy* or girl*).ti,ab,jw,nw.
10. (schoolchild$ or "school age" or "school aged" or preschool$ or toddler$ or kid? or kindergar$ or boy? or girl?).ti,ab,jw,nw.
11. or/5-10
12. TERMINALLY ILL/
13. ((terminal$ or final or advance$ or incurable or life limit$) adj3 (ill$ or disease$ or condition$)).ti,ab.
14. dying.mp. or cancer.ti,ab. [mp=title, abstract, original title, name of substance word, subject heading word, floating sub-heading word, keyword heading word, protocol supplementary concept word, rare disease supplementary concept word, unique identifier, synonyms]
15. (end adj3 life).ti,ab.
16. ((approach$ or close$ or near$ or imminent$ or impending) adj3 death).ti,ab.
17. (Body adj2 (shut? down or shutting down or deteriorat$)).ti,ab.
18. (deathbed? or death bed? or passing away or passing on or expiring or expiration).ti,ab.
19. ((last or final) adj1 (hour$ or days$ or minute$)).ti,ab.
20. (last year of life or LYOL or life$ end).ab,ti.
21. (advance$ stage? or final stage? or end stage? or last stage? or late stage? or terminal stage?).ti,ab.
22. ((advanced or late or last or end or final or terminal) adj phase$).ab,ti.
23. TERMINAL CARE/
24. (terminal$ adj3 (care$ or caring)).ti,ab.
25. PALLIATIVE CARE/
26. palliat$.ti,ab.
27. HOSPICE CARE/
28. hospice?.ab,ti.
29. or/12-28
30. exp pain assessment/
31. (VAS or NRS or VRS).mp.
32. (visual adj2 analog* adj2 (scale* or score*)).mp.
33. (numeric* adj2 rat* adj2 (scale* or score*)).mp.
34. (verbal* adj2 rat* adj2 (scale* or score*)).mp.
35. (faces adj2 (scale* or score*)).mp.
36. (piece* adj2 hurt adj2 tool*).mp.
37. oucher.mp.
38. FLACC.mp.
39. (Face* adj2 Leg* adj2 Activit* adj2 Cry adj2 Consol*).mp.
40. (Analog* adj2 Chromatic adj2 Continu* adj2 (scale* or score*)).mp.
41. body outline.mp.
42. (pain* adj2 (scale* or score*)).mp.
43. (child* adj2 global adj2 rat* adj2 (scale* or score*)).mp.
44. (colo?r adj2 analog* adj2 (scale* or score*)).mp.
45. (eland adj2 colo?r adj2 tool*).mp.

46. (facial adj2 express* adj2 (scale* or score*)).mp.
47. (glass* adj2 rat* adj2 (scale* or score*)).mp.
48. (poker adj2 chip adj2 tool*).mp.
49. (pain* adj2 ladder*).mp.
50. (Scheffield* adj6 pain* adj2 tool*).mp.
51. (Smiley adj2 analog* adj2 scale*).mp.
52. (4-point* adj2 verbal adj2 descript* adj2 scale*).mp.
53. (four-point* adj2 verbal adj2 descript* adj2 scale*).mp.
54. (visual adj2 analog* adj2 toy*).mp.
55. (5-point* adj2 word* adj2 graphic* adj6 scale*).mp.
56. (five-point* adj2 word* adj2 graphic* adj6 scale*).mp.
57. (6-point* adj2 word* adj2 graphic* adj6 scale*).mp.
58. (six-point* adj2 word* adj2 graphic* adj6 scale*).mp.
59. MSPCT.mp.
60. 4-VDS.mp.
61. VNRS.mp.
62. VASOF.mp.
63. (5-WGRS or 6-WGRS).mp.
64. or/30-63
65. (instrumentation or methods).sh.
66. (Validation Studies or Comparative Study).pt.
67. exp Psychometrics/
68. psychometr*.ti,ab.
69. (clinimetr* or clinometr*).tw.
70. Outcome Assessment/
71. outcome assessment.ti,ab.
72. outcome measure*.tw.
73. exp Observer Variation/
74. observer variation.ti,ab.
75. exp Health Status Indicators/
76. exp Reproducibility of Results/
77. reproducib*.ti,ab.
78. exp Discriminant Analysis/
79. (reliab* or unreliab* or valid* or coefficient or homogeneity or homogeneous or internal consistency).ti,ab.

80. (cronbach* and (alpha or alphas)).ti,ab.
81. (item and (correlation* or selection* or reduction*)).ti,ab.
82. (agreement or precision or imprecision or precise values or test-retest).ti,ab.
83. (reliab* and (test or retest)).ti,ab.
84. (test and retest).ti,ab.
85. (stability or interrater or inter-rater or intrarater or intra-rater or intertester or inter-tester or intratester or intra-tester or interobserver or inter-observer or intraobserver or intraobserver or intertechnician or inter-technician or intratechnician or intra-technician or interexaminer or inter-examiner or intraexaminer or intra-examiner or interassay or interassay or intraassay or intra-assay or interindividual or inter-individual or intraindividual or intra-individual or interparticipant or inter-participant or intraparticipant or intra-participant or kappa* or repeatab*).ti,ab.
86. ((replicab* or repeated) and (measure or measures or findings or result or results or test or tests)).ti,ab.

87. (generaliza* or generalisa* or concordance).ti,ab.
88. (intraclass and correlation*).ti,ab.
89. (discriminative or known group or factor analysis or factor analyses or dimension* or subscale*).ti,ab.
90. (multitrait and scaling and (analysis or analyses)).ti,ab.
91. (item discriminant or interscale correlation* or error or errors or individual variability).ti,ab.
92. (variability and (analysis or values)).ti,ab.
93. (uncertainty and (measurement or measuring)).ti,ab.
94. (standard error of measurement or sensitiv* or responsive*).ti,ab.

95. ((minimal or minimally or clinical or clinically) and (important or significant or detectable) and (change or difference)).ti,ab.
96. (small* and (real or detectable) and (change or difference)).ti,ab.
97. (meaningful change or ceiling effect or floor effect or Item response model or IRT or Rasch or Differential item functioning or DIF or computer adaptive testing or item bank or cross-cultural equivalence).ti,ab.
98. or/65-97
99. ANIMALS/ not HUMANS/
100. exp ANIMALS, LABORATORY/
101. exp ANIMAL EXPERIMENTATION/
102. exp MODELS, ANIMAL/
103. exp RODENTIA/
104. (rat or rats or mouse or mice).ti.
105. or/99-104
106. (4 and 11 and 29 and 64 and 98) not 105

EMBASE via Ovid

1. exp Pain/
2. Pain Management/
3. (pain* or headache* or migraine* or neuralgia or neuropathic or myalgia or metatarsalgia or sciatica).ab,ti.
4. or/1-3
5. (infan* or newborn* or new-born* or neonat* or baby or babies or child* or youth or kid or kids or toddler* or boy* or girl* or adolescen* or teen* or juvenile* or p?ediatric*).ti,ab,jw.
6. *TERMINALLY ILL/
7. ((terminal$ or final or advance$ or incurable or life limit$) adj3 (ill$ or disease$ or condition$)).ti,ab.
8. (dying or cancer).ti,ab.
9. (end adj3 life).ti,ab.
10. ((approach$ or close$ or near$ or imminent$ or impending) adj3 death).ti,ab.
11. (Body adj2 (shut? down or shutting down or deteriorat$)).ti,ab.
12. (deathbed? or death bed? or passing away or passing on or expiring or expiration).ti,ab.
13. ((last or final) adj1 (hour$ or days$ or minute$)).ti,ab.
14. (last year of life or LYOL or life$ end).ab,ti.
15. (advance$ stage? or final stage? or end stage? or last stage? or late stage? or terminal stage?).ti,ab.
16. ((advanced or late or last or end or final or terminal) adj phase$).ab,ti.
17. *TERMINAL CARE/
18. (terminal$ adj3 (care$ or caring)).ti,ab.
19. *PALLIATIVE CARE/
20. palliat$.ti,ab.
21. *HOSPICE CARE/
22. hospice?.ab,ti.
23. or/6-22
24. exp pain assessment/
25. (VAS or NRS or VRS).mp.
26. (visual adj2 analog* adj2 (scale* or score*)).mp.
27. (numeric* adj2 rat* adj2 (scale* or score*)).mp.
28. (verbal* adj2 rat* adj2 (scale* or score*)).mp.
29. (faces adj2 (scale* or score*)).mp.
30. (piece* adj2 hurt adj2 tool*).mp.
31. (piece* adj2 hurt adj2 tool*).mp.
32. FLACC.mp.
33. (Face* adj2 Leg* adj2 Activit* adj2 Cry adj2 Consol*).mp.
34. (Analog* adj2 Chromatic adj2 Continu* adj2 (scale* or score*)).mp.
35. body outline.mp.
36. (pain* adj2 (scale* or score*)).mp.
37. (child* adj2 global adj2 rat* adj2 (scale* or score*)).mp.
38. (colo?r adj2 analog* adj2 (scale* or score*)).mp.
39. (eland adj2 colo?r adj2 tool*).mp.
40. (facial adj2 express* adj2 (scale* or score*)).mp.
41. (glass* adj2 rat* adj2 (scale* or score*)).mp.
42. (poker adj2 chip adj2 tool*).mp.
43. (pain* adj2 ladder*).mp.
44. (Scheffield* adj6 pain* adj2 tool*).mp.
45. (Smiley adj2 analog* adj2 scale*).mp.
46. (4-point* adj2 verbal adj2 descript* adj2 scale*).mp.
47. (four-point* adj2 verbal adj2 descript* adj2 scale*).mp.
48. (visual adj2 analog* adj2 toy*).mp.
49. (5-point* adj2 word* adj2 graphic* adj6 scale*).mp.
50. (five-point* adj2 word* adj2 graphic* adj6 scale*).mp.
51. (6-point* adj2 word* adj2 graphic* adj6 scale*).mp.
52. (six-point* adj2 word* adj2 graphic* adj6 scale*).mp.

53. MSPCT.mp.
54. 4-VDS.mp.
55. VNRS.mp.
56. VASOF.mp.
57. (5-WGRS or 6-WGRS).mp.
58. or/24-56
59. exp intermethod comparison/ or exp data collection method/ or validation study.mp. or exp feasibility study/ or exp pilot study/ or exp psychometry/ or exp reproducibility/ or reproducib*.ab,ti. or audit.ab,ti. or psychometr*.ab,ti. or clinimetr*.ab,ti. or clinometr*.ab,ti. or observer variation expOR observer variation.ab,ti. or discriminant analysis expOR validity/exp or reliab*.ab,ti. or valid*.ab,ti. or coefficient.ab,ti. or internal consistency.ab,ti. or (cronbach* and (alpha or alphas)).ab,ti. or item correlation.ab,ti. or item correlations.ab,ti. or item selection.ab,ti. or item selections.ab,ti. or item reduction.ab,ti. or item reductions.ab,ti. or agreement.ab,ti. or precision.ab,ti. or imprecision.ab,ti. or precise values.ab,ti. or test-retest.ab,ti. or (test and retest).ab,ti. or (reliab* and (test or retest)).ab,ti. or stability.ab,ti. or interrater.ab,ti. or inter-rater.ab,ti. or intrarater.ab,ti. or intra-rater.ab,ti. or intertester.ab,ti. or inter-tester.ab,ti. or intratester.ab,ti. or intratester.ab,ti. or interobeserver.ab,ti. or inter-observer.ab,ti. or intraobserver.ab,ti. or intraobserver.ab,ti. or intertechnician.ab,ti. or inter-technician.ab,ti. or intratechnician.ab,ti. or intratechnician.ab,ti. or interexaminer.ab,ti. or inter-examiner.ab,ti. or intraexaminer.ab,ti. or intraexaminer.ab,ti. or interassay.ab,ti. or inter-assay.ab,ti. or intraassay.ab,ti. or intra-assay.ab,ti. or interindividual.ab,ti. or inter-individual.ab,ti. or intraindividual.ab,ti. or intra-individual.ab,ti. or interparticipant.ab,ti. or inter-participant.ab,ti. or intraparticipant.ab,ti. or intraparticipant.ab,ti. or kappa.ab,ti. or kappas.ab,ti. or coefficient of variation.ab,ti. or repeatab*.ab,ti. or ((replicab* or repeated) and (measure or measures or findings or result or results or test or tests)).ab,ti. or generaliza*.ab,ti. or generalisa*.ab,ti. or concordance.ab,ti. or (intraclass and correlation*).ab,ti. or discriminative.ab,ti. or known group.ab,ti. or factor analysis.ab,ti. or factor analyses.ab,ti. or factor structure.ab,ti. or factor structures.ab,ti. or dimensionality.ab,ti. or subscale*.ab,ti. or multitrait scaling analysis.ab,ti. or multitrait scaling analyses.ab,ti. or item discriminant.ab,ti. or interscale correlation.ab,ti. or interscale correlations.ab,ti. or ((error or errors) and (measure* or correlat* or evaluat* or accuracy or accurate or precision or mean)).ab,ti. or individual variability.ab,ti. or interval variability.ab,ti. or rate variability.ab,ti. or variability analysis.ab,ti. or (uncertainty and (measurement or measuring)).ab,ti. or standard error of measurement.ab,ti. or sensitiv*.ab,ti. or responsive*.ab,ti. or (limit and detection).ab,ti. or minimal detectable concentration.ab,ti. or interpretab*.ab,ti. or (small* and (real or detectable) and (change or difference)).ab,ti. or meaningful change.ab,ti. or minimal important change.ab,ti. or minimal important difference.ab,ti. or minimally important change.ab,ti. or minimally important difference.ab,ti. or minimal detectable change.ab,ti. or minimal detectable difference.ab,ti. or minimally detectable change.ab,ti. or minimally detectable difference.ab,ti. or minimal real change.ab,ti. or minimal real difference.ab,ti. or minimally real change.ab,ti. or minimally real difference.ab,ti. or ceiling effect.ab,ti. or floor effect.ab,ti. or item response model.ab,ti. or irt.ab,ti. or rasch.ab,ti. or differential item functioning.ab,ti. or dif.ab,ti. or computer adaptive testing.ab,ti. or item bank.ab,ti. or cross-cultural equivalence.ab,ti.
60. ANIMAL/ not HUMAN/
61. NONHUMAN/
62. exp ANIMAL EXPERIMENT/
63. exp EXPERIMENTAL ANIMAL/
64. ANIMAL MODEL/
65. exp RODENT/
66. (rat or rats or mouse or mice).ti.
67. or/59-65
68. (4 and 5 and 23 and 57 and 58) not 66

CINAHL® database (Cumulative Index to Nursing and Allied Health Literature) via EBSCOhost

Search ID#	**Search Terms**
S1	(MH "Pain+") OR (MH "Pain Management") OR (TI (pain* or headache* or migraine* or neuralgia or neuropathic))
S2	(MH "Child, Preschool") OR (MH "Adolescence+") OR (MH "Child+") OR (MH "Infant+") OR (MH "Infant, Newborn") OR TX(infan* or newborn* or new-born* or neonat* or baby or babies or child* or youth or kid or kids or toddler* or boy* or girl* or adolescen* or teen* or juvenile* or pediatric* or paediatric*)
S3	TI ((terminal* or final or advance* or incurable or "life limit*") adj3 (ill* or disease* or condition*)) OR dying OR cancer OR (end adj3 life) OR ((approach* or close* or near* or imminent* or impending) adj3 death) OR (Body adj2 ("shut* down" or "shutting down" or deteriorat*)) OR (deathbed* or "death bed*" or "passing away" or "passing on" or expiring or expiration).ti,ab. 20 ((last or final) adj1 (hour* or days* or minute*)) OR (last year of life or LYOL or life* end) OR (advance* stage* or final stage* or end stage* or last stage* or late stage* or terminal stage*) OR ((advanced or late or last or end or final or terminal) adj phase*) OR TERMINAL CARE/ OR (terminal* adj3 (care* or caring)) OR PALLIATIVE CARE/ OR palliat* OR HOSPICE CARE/ OR hospice*)
S4	MH "Pain Measurement"<br>OR TX((pain AND (tool OR tools OR instrument OR instruments OR measure OR measures OR measurement OR scale OR scales OR observational OR observation OR behavioral OR behavioural OR biobehavioral OR assess OR assessment OR test OR tests)) OR ((MH "Clinical Assessment Tools+") AND ((MH "Pain+") OR "pain"))<br>OR<br>TX ((5-WGRS or 6-WGRS)<br>OR VASOF<br>OR VNRS<br>OR 4-VDS<br>OR MSPCT<br>OR (six-point* N2 word* N2 graphic* N6 scale*)<br>OR (6-point* N2 word* N2 graphic* N6 scale*)<br>OR (five-point* N2 word* N2 graphic* N6 scale*)<br>OR (5-point* N2 word* N2 graphic* N6 scale*)<br>OR (visual N2 analog* N2 toy*)<br>OR (four-point* N2 verbal N2 descript* N2 scale*)<br>OR (4-point* N2 verbal N2 descript* N2 scale*)<br>OR (Smiley N2 analog* N2 scale*)<br>OR (Scheffield* N6 pain* N2 tool*)<br>OR (pain* N2 ladder*)<br>OR (poker N2 chip N2 tool*)<br>OR (glass* N2 rat* N2 (scale* or score*))<br>OR (facial N2 express* N2 (scale* or score*))<br>OR (eland N2 (color or colour) N2 tool*)<br>OR ((color or colour) N2 analog* N2 (scale* or score*))<br>OR (child* N2 global N2 rat* N2 (scale* or score*))<br>OR (pain* N2 (scale* or score*))<br>OR body outline<br>OR (Analog* N2 Chromatic N2 Continu* N2 (scale* or score*))<br>OR (Face* N2 Leg* N2 Activit* N2 Cry N2 Consol*)<br>OR FLACC<br>OR oucher<br>OR (piece* N2 hurt N2 tool*)<br>OR (faces N2 (scale* or score*))<br>OR (verbal* N2 rat* N2 (scale* or score*))<br>OR (numeric* N2 rat* N2 (scale* or score*))

OR (visual N2 analog* N2 (scale* or score*))
OR (VAS or NRS or VRS))

S5 (MH “Psychometrics”) or (TI psychometr* or AB psychometr*) or (TI clinimetr* or AB clinimetr*) or (TI clinometr* OR AB clinometr*) or (MH “Outcome Assessment”) or (TI outcome assessment or AB outcome assessment) or (TI outcome measure* or AB outcome measure*) or (MH “Health Status Indicators”) or (MH “Reproducibility of Results”) or (MH “Discriminant Analysis”) or ((TI reproducib* or AB reproducib*) or (TI reliab* or AB reliab*) or (TI unreliab* or AB unreliab*)) or ((TI valid* or AB valid*) or (TI coefficient or AB coefficient) or (TI homogeneity or AB homogeneity)) or (TI homogeneous or AB homogeneous) or (TI “coefficient of variation” or AB “coefficient of variation”) or (TI “internal consistency” or AB “internal consistency”) or (MH “Internal Consistency+”) or (MH “Reliability+”) or (MH “Measurement Error+”) or (MH “Content Validity+”) or “hypothesis testing” or “structural validity” or “cross-cultural validity” or (MH “Criterion-Related Validity+”) or “responsiveness” or “interpretability” or (TI reliab* or AB reliab*) and ((TI test or AB test) OR (TI retest or AB retest)) or (TI stability or AB stability) or (TI interrater or AB interrater) or (TI inter-rater or AB inter-rater) or (TI intrarater or AB intrarater) or (TI intra-rater or AB intrarater) or (TI intertester or AB intertester) or (TI inter-tester or AB inter-tester) or (TI intratester or AB intratester) or (TI intra-tester or AB intra-tester) or (TI interobserver or AB interobserver) or (TI inter-observer or AB inter-observer) or (TI intraobserver or AB intraobserver) or (TI intra-observer or AB intra-observer) or (TI intertechnician or AB intertechnician) or (TI inter-technician or AB inter-technician) or (TI intratechnician or AB intratechnician) or (TI intra-technician or AB intra-technician) or (TI interexaminer or AB interexaminer) or (TI inter-examiner or AB inter-examiner) or (TI intraexaminer or AB intraexaminer) OR (TI intra-examiner or AB intra-examiner) or (TI intra-examiner or AB intraexaminer) or (TI interassay or AB interassay) or (TI inter-assay or AB inter-assay) or (TI intraassay or AB intraassay) or (TI intra-assay or AB intra-assay) or (TI interindividual or AB interindividual) or (TI inter-individual or AB inter-individual) OR (TI intraindividual or AB intraindividual) or (TI intra-individual or AB intra-individual) or (TI interparticipant or AB interparticipant) or (TI inter-participant or AB inter-participant) or (TI intraparticipant or AB intraparticipant) or (TI intra-participant or AB intra-participant) or (TI kappa or AB kappa) or (TI kappa’s or AB kappa’s) or (TI kappas or AB kappas) or (TI repeatab* or AB repeatab*) or (TI responsive* or AB responsive*) or (TI interpretab* or AB interpretab*)

S6 labour OR labor OR childbirth OR birth OR postpartum

S7 S1 AND S2 AND S3 AND S4 AND S5

S8 S7 not S6

S9 S7 not S6
Narrow by SubjectAge: - infant, newborn: birth-1 month
Narrow by SubjectAge: - infant: 1-23 months
Narrow by SubjectAge: - all infant
Narrow by SubjectAge: - child, preschool: 2-5 years
Narrow by SubjectAge: - child: 6-12 years
Narrow by SubjectAge: - adolescent: 13-18 years
Narrow by SubjectAge: - all child

PsycInfo via ProQuest

1. pain assessment
2. pain measurement
3. 1 or 2

4. validity
5. classical test theory
6. psychometr*
7. Rasch
8. “differential item functioning”
9. DIF
10. known group$
11. confirmatory factor analysis
12. exploratory factor analysis
13. principal components analysis
14. reliability
15. reproducibility
16. test-retest
17. intra-rater
18. internal consistency
19. split-half
20. inter-item
21. equivalence
22. (alternative or parallel or equivalent) and forms
23. responsiveness
24. interpretability
25. feasibility
26. clinical acceptability
27. 4 or 5 or 6 or 7 or 8 or 9 or 10 or 11 or 12 or 13 or 14 or 15 or 16 or 17 or 18 or 19 or 20 or 21 or 22 or 23 or 24 or 25 or 26

28. paediatric
29. child*
30. neonat*
31. adolescent*
32. pediatric*
33. paediatric*
34. 28 or 29 or 30 or 31 or 32 or 33

35. palliative*
36. terminal*
37. dying
38. “end of life”
39. EOL
40. limited survival
41. hospice
42. tumor*
43. cancer

44. neoplasm
45. 35 or 36 or 37 or 38 or 39 or 40 or 41 or 42 or 43 or 44 or 45

46. 3 and 27 and 34 and 45
47. Limited by: Date: From April 01 2006 to September 30 2018; Language:English; Age group:Adolescence (13-17 Yrs), Preschool Age (2-5 Yrs), School Age (6-12 Yrs); Population:Human

Web of Science Core Collection
1. pain assessment
2. pain measurement
3. #2 OR #1
4. validity
5. classical test theory
6. psychometr*
7. Rasch
8. “differential item functioning”
9. DIF
10. known group$
11. confirmatory factor analysis
12. exploratory factor analysis
13. principal components analysis
14. reliability
15. reproducibility
16. test-retest
17. intra-rater
18. internal consistency
19. split-half
20. inter-item
21. equivalence
22. (alternative or parallel or equivalent) and forms
23. responsiveness
24. interpretability
25. feasibility
26. clinical acceptability
27. #26 OR #25 OR #24 OR 23 OR #22 OR #21 OR #20 OR #19 OR #18 OR #17 OR #16 OR #15 OR #14 OR #13 OR #12 OR #11 OR #10 OR #9 OR #8 OR #7 OR #6 OR #5 OR #4

28. child
29. neonat*
30. adolescen*
31. paediatric
32. paediatrics
33. pediatric
34. pediatrics
35. #34 OR #33 OR #32 OR #31 OR #30 OR #29 OR #28

36. palliative*
37. terminal*
38. dying
39. “end of life”
40. EOL
41. limited survival
42. hospice
43. tumor*
44. cancer

45. neoplasm
46. #45 OR #44 OR #43 OR #42 OR #41 OR #40 OR #39 OR #38 OR #37 OR #36
47. #46 AND #35 AND #27 AND #3
48. #42 AND #31 AND #23 AND #3 Refined by: PUBLICATION YEARS: (2018 OR 2012 OR 2006 OR 2017 OR 2011 OR 2016 OR 2010 OR 2015 OR 2009 OR 2014 OR 2008 OR 2013 OR 2007) AND LANGUAGES: (ENGLISH)
