# Supplementary material for: Pain assessment tools in paediatric palliative care: A systematic review of psychometric properties and recommendations for clinical practice
Source: Palliat Med. 2021 Dec 29;36(1):30–43. doi: 10.1177/02692163211049309 (PMC8796159; doi:10.1177/02692163211049309)
Supplement: sj-pdf-2-pmj-10.1177_02692163211049309 – Supplemental material for Pain assessment tools in paediatric palliative care: A systematic review of psychometric properties and recommendations for clinical practice [file sj-pdf-2-pmj-10.1177_02692163211049309.pdf]

Supplementary Table 2. Methodological Quality and Measurement Property Results

A. Content validity and internal structure

Measure	Country (language)	age group	Content validity				Structural validity (% variance explained)				Cross-cultural validity				Internal consistency			
			n	Result	Rating	Meth qual	n	Result	Rating	Meth qual	n	Result (rating)	Rating	Meth qual	n	Result (Cronbach alpha coefficient)	Rating	Meth qual
APPT	Portugal (Portuguese) (Fernandes et al., 2015)	8-17	24 children	Relevance and comprehensibility (patients): assessed with the Q-sort method, 58 words were known by >75% of children, comprehensiveness not assessed.	+	Doubtful					3 Portuguese native reviewers; 2 English native translators	independent translation, back-translation	?	Inadequate				
	Turkey (Turkish) (Özalp Gerçeker et al., 2018)	8-17	10 paediatric pain experts	Relevance (expert): Content validity index (CVI) = 0.8-1.0 (+)	+	Adequate					5 language experts	independent translation, back-translation, experts committee were used to provide cross-cultural validity in the development process	+	Doubtful	96	APPT-T α=0.78	+	Adequate
			10 children or adolescents with cancer	Relevance and comprehensibility (patients): assessed with the Q-sort method, 89% of 63 words categorised as "I use this word to describe hurt/ pain" by participants, comprehensiveness not assessed.	+	Doubtful												
COMFORT	United States (English) (Ambuel et al., 1992)	n.r.	20 experienced clinicians	Experts were only involved before development of scale, relevance and comprehensibility were not explicitly consulted, patients were not involved in content validation.	+	Inadequate	50	PCA: Dimension 1 & 2 (84%) Dimension 1 (58%) Dimension 2 (26%)	+	Adequate					50	α=0.90	+	Very good
	The Netherlands (English) (van Dijk et al., 2000)	0-3													158	α=0.90, 0.92, 0.92	+	Very good
CPI	United States (English/ Spanish determined by language use) (Pfefferbaum et al., 1990, Adams, 1988, Adams, 1989)	3-15.9													35	α=0.80 in Anglo children	+	Adequate
															43	α=0.72 in Hispanic children	+	
DEGR	France (French)	2-6					80	Factor 1 (51%) Factor 2 (13,14%) Factor 3 (8, 10%)	+	Doubtful								

	(Gauvain-Piquard et al., 1987)																	
	France (French)<br><br>(Gauvain-Piquard et al., 1999)	2-6					152	Factor 1 (50%)<br>Factor 2 (15%)	+	Adequate					152	α=0.90, 0.93, 0.84, 0.74 respectively for total score, pain subscale, psychomotor inertia, and anxiety subscale	+	Very good
	France (French)<br><br>(Marec-Berard et al., 2015)	2-6					59	factorial analysis, correlation coefficients, between score of each item and total scores of other items >0.5 for 7/10 items	+	Inadequate					59	α = 0.845	+	Adequate
							48	factorial analysis, correlation coefficients, between score of each item and total scores of other items >0.5 for 4/10 items	-	Inadequate					48	α = 0.777	+	Adequate
DOLLS																		
FLACC	Brazil (Brazilian Portguese)<br><br>(Da Silva et al., 2011)	7-17													90	α=0.76	+	Adequate
Le Baron and Zeltzer, 1984 / Kuttner and LePage, 1989 Faces Scale	United States (English/ Spanish determined by language use)<br><br>(Pfefferbaum et al., 1990, Adams, 1988, Adams, 1989)	3-15.9													35	α=0.85	+	Adequate
															43	α=0.87	+	Adequate
FPS-R	Spain (Catalan)<br><br>(Miro and Huguet, 2004)	7-15									1 bilingual psychologist 1 native English speaker	independent translation and back-translation were used to provide cross-cultural validity in the development process	+	Doubtful				
HEDEN	France (French)<br><br>(Marec-Berard et al., 2015)	2-6													59	α = 0.61 (95% CI: 0.48–0.79)	-	Adequate
															48	α = 0.75 (95% CI: 0.48–0.79)	-	Adequate
MSAS (7-12)	United Kingdom (English)<br><br>(Collins et al., 2002)	7-12													149	α = 0.67	-	Adequate

OMDQ																		
OPS																		
Pain Squad	Canada (English)<br><br>(Stinson et al., 2015)	8-18	10 paediatric oncologists<br>10 paediatric pain experts	Development (expert): expert opinion was used to develop the tool (+)	+	Doubtful									92	8-18 (N=92)<br>α = 0.96	+	Adequate
			18 adolescents with cancer	Relevance (patients): 88% of questions rated as "important" or "very important" by >50% of adolescents (+)	+	Doubtful												
PBCL	United States (English/ Spanish)<br><br>(Pfefferbaum et al., 1990)	3-15.9													35	α=0.78 in Anglo children	+	Adequate
															43	α=0.54 in Hispanic children	-	Adequate
PCT																		
PII	United States (English)<br><br>(Martin et al., 2015)	6.6-24.1													60	α = 0.84	+	Adequate
PII-P	United States (English)<br><br>(Martin et al., 2015)	6.6-24.1													65	α = 0.94 (mothers) and 0.96 (fathers)	+	Adequate
PPP	Brazil (Brazilian Portuguese)<br><br>(Pasin et al., 2013)	1-18	20 health professionals<br>10 primary caregivers/ parents	Clarity:<br>median between 3.00 (clear) and 4.00 (very clear); mean= 3.49, SD = 0.82) (+)	+	Inadequate					2 bilingual translators; 2 English translators; 2 registered nurses; 2 doctors; 2 physiotherapists; 1 pain specialist; 1 teacher; researchers; author of original version	independent translation; back-translation; experts committee: experts maintain consensus and semantic equivalence	+	Adequate	45	α = 0.864	+	Adequate
	United Kingdom (English)<br><br>(Hunt et al., 2007)	1-18													29	α = 0.88	+	Adequate
	United Kingdom (English)	1-18													140	α = 0.75-0.89	+	Adequate

	(Hunt et al., 2004)						
RPS	Canada (English)<br><br>(Mahon et al., 2015)	5-10					
SSPedi	Canada and United States (English)<br><br>(Dupuis et al., 2018)	8-18.7		502	α = 0.86	+	Adequate
WBS							

APPT - Adolescent Paediatric Pain Tool; CPI - Children's Procedural Interview; DEGR scale - Douleur Enfant Gustave Roussy; FPS-R – Faces Pain Scale-Revised; FLACC scale - Face, Legs, Activity, Cry, Consolability scale; HEDEN scale - Hétero Evaluation Douleur Enfant scale; MIPS - Modified Infant Pain Scale; MSAS - Memorial Symptom Assessment Scale; OMDQ - Oral Mucositis Daily Questionnaire; OPS - Objective Pain Scale; PBCL - Pain Behaviour Check List; PCT - Poker Chip Tool; PII - Pain Interference Index; PII-P - Pain Interference Index-Parent; PPP - Paediatric Pain Profile; r - Pearson product moment correlation coefficient; RPS - Rainbow Pain Scale; rs - Spearman correlation coefficient; SSPedi - Symptom Screening in Paediatrics; τ - Kendall's tau (τ) correlation coefficient; WBS - Wong-Baker FACES Pain Rating Scale

B. Other measurement properties.

Measure	Country (language) in which the questionnaire was evaluated	Hypothesis testing						Criterion validity					Reliability			
		age group	used reference (reported by)	n	Convergent/ Divergent validity: Result	Rating	Meth qual	used reference (reported by)	n	Predictive/ concurrent validity: Result	Rating	Meth qual	N	Results	Rating	Meth qual
APPT	Turkey (Turkish) (Özalp Gerçeker et al., 2018)	8-17											30	**test-retest:** ICC = 0.84 (total number of body areas marked), 0.73 (pain severity), 0.72 (pain intensity ratings), and 0.82 (total number of word descriptors). No significant differences at repeated measurements (p>0.05) for all sections	+	Adequate
COMFORT	United States (English) (Ambuel et al., 1992)	n.r.	VAS (clinicians)	50	**Convergent validity: Significant** r=0.75, P<0.01	+	Very good						50	**Interrater agreement:** r = 0.84, p <0.01	+	Doubtful
	The Netherlands (English) (van Dijk et al., 2000)	0-3	VAS (nurses)	26	**Convergent validity:** before r = 0.64-0.73; after r = 0.79-0.83	+	Very good						158	**Interrater agreement:** K=0.70 (0.63-0.93)	+	Adequate
	The Netherlands (English) (Van Dijk et al., 2001)	0-3	HRV	158	**Convergent validity: Significant** r=0.44, 0.31-0.55	-	Adequate									
			HRV		**Convergent validity: Significant** r=0.48, 0.35-0.58	-	Adequate									
			MAP		**Convergent validity: Significant** r=0.0.37, 0.24-0.49	-	Adequate									
			MAPV		**Convergent validity: Significant** r=0.49, 0.36-0.59	-	Adequate									
CPI	United States (English/ Spanish determined by language use) (Pfefferbaum et al., 1990, Adams, 1988, Adams, 1989)	3-15.9	PBCL	35	**Convergent validity:** r=0.66, P<0.001 in Anglo children	-	Very good									
				43	**Convergent validity**: r=0.64, P<0.001 in Hispanic children	-	Very good									
			Age	35	**Divergent validity: significant** r =-0.40, p<0.01 in Anglo children	-	Very good									
				43	**Divergent validity: significant** r=-0.46, p<0.001 in Hispanic children	-	Very good									
			Gender	35	**Divergent validity: Not significant** r =not reported in Anglo children	-	Very good									
				43	**Divergent validity: Not significant** r=not reported in Hispanic children	-	Very good									
			Duration and stage of illness	35	**Divergent validity: Not significant** r =not reported in Anglo children	-	Very good									
				43	**Divergent validity: Not significant** r=not reported in Hispanic children	-	Very good									

			Parent anxiety	35	**Divergent validity: Significant**<br>r =0.51, p<0.01 in Anglo children	-	Very good									
				43	**Divergent validity: Not significant**<br>r=not reported in Hispanic children	-	Very good									
DEGR	France (French)<br><br>(Gauvain-Piquard et al., 1987)	2-6											80	Interrater agreement:<br>K=0.14-0.60, significantly different from 0, <0.7; agreement was better for auxillaries (0.32-0.60) than nurses (0.14-0.53)	-	Very good
	France (French)<br><br>(Gauvain-Piquard et al., 1999)	2-6	pain specialists' ratings	53	**Convergent validity:**<br>r = 0.74-0.87, p<0.001	+	Very good						152	Interrater agreement:<br>significant weighted kappa coefficients for all items, p<0.001, varied from 0.28-0.45, weighted kappa coefficient for total DEGRR score = 0.71> 0.7	+	Very good
			gender	152	**Divergent validity: Not significant**<br>r = not reported, p >0.05	-	Very good									
			age	152	**Divergent validity: Not significant**<br>r = not reported, p >0.05	-	Very good									
			psychosocial characteristics	152	**Divergent validity: Not significant**<br>r = not reported, p >0.05	-	Very good									
			medical characteristics	152	**Divergent validity: Not significant**<br>r = not reported, p >0.05	-	Very good						53	Interspecialist agreement: K=0.4 (4 specialists)	-	Doubtful
	France (French)<br><br>(Marec-Berard et al., 2015)	2-6	HEDEN	59	**Convergent validity:**<br>r = 0.50	-	Very good									
			HEDEN	48	**Convergent validity:**<br>r = 0.6	-	Very good									
DOLLS	Lebanon (Arabic)<br><br>(Badr Zahr et al., 2006)	4-10	Scores before procedure	45	**Divergent validity: Significant**<br>t = 12.45, p<0.01	+	Very good	WBS	45	**Concurrent validity: Significant**<br>r = 0.90, p<0.01	+	Very good				
			heart rate	45	**Convergent validity: Significant**<br>r = 0.78, 0.89, 0.74, p<0.001	+	Very good									
			systolic blood pressure	45	**Convergent validity: Significant**<br>r = 0.75, 0.81, 0.79 p<0.001	+	Very good									
			oxygen saturation	45	**Convergent validity: no correlation**<br>DOLLS and are not correlated	-	Very good									
			OSBD-R (parents)	45	**Convergent validity: Significant**<br>r = 0.76, p<0.01	+	Very good									
			WBS (parents)	45	**Convergent validity: Significant**<br>r = 0.81, p<0.01	+	Very good									
			FLACC (nurses)	45	**Convergent validity: Significant**<br>r = 0.82, p<0.01	+	Very good									
			OSBD-R (nurses)	45	**Convergent validity: Significant**<br>r = 0.80, p<0.01	+	Very good									
FLACC	United States (English)	<1-7	postanalgesia scores	89	**Divergent validity: Significant**<br>Preanalgesia FLACC scores (7.0+/-2.9) are significantly higher than that postanalgesia at 10, 30, 60: (1.7	+	Adequate						89	Interrater agreement:<br>r = 0.94, kappa value >0.5;<br>face: kappa = 0.52;<br>legs: kappa = 0.67;	+	Very good

	(Merkel et al., 1997)				+/- 2.2), (1.0 +/- 1.9),(0.2 +/- 0.5) , p<0.001									activity: kappa = 0.72<br>cry: kappa = 0.82;<br>consolability: kappa = 0.66		
			pain global ratings (nurses)	89	**Convergent validity: Significant**<br>r = 0.41 p<0.005	-	Very good									
			OPS	89	**Convergent validity: Significant**<br>r = 0.80 p<0.001	+	Very good									
	Lebanon (Arabic)<br><br>(Badr Zahr et al., 2006)	4-10	OSBD-R (parents)	45	**Convergent validity: Significant**<br>r = 0.89	+	Very good									
			DOLLS (self)	45	**Convergent validity: Significant**<br>r = 0.82	+	Very good	DOLLS	45	**Concurrent validity: Significant**<br>r = 0.82, p<0.01	+	Very good				
			FACES (self)	45	**Convergent validity: Significant**<br>r = 0.78	+	Very good									
	United States (English)<br><br>(Manworren and Hynan, 2003)	<3	postanalgesia scores	147	**Divergent validity: Significant**<br>Preanalgesia (Time 1) FLACC scores, 95%CI (7.0, 6.66-7.41) significantly higher than postanalgesia Time 2 (2.05, 1.68-2.43) , Time 3 (0.74,0.48-1.00)	+	Very good									
	Brazil (Brazilian Portuguese)<br><br>(Da Silva et al., 2011)	7-17	gender	90	**Divergent validity: Not significant**<br>FLACC-B r = not reported, p =0.36	-	Very good									
Le Baron and Zeltzer, 1984 / Kuttner and LePage, 1989 Faces Scale	United States (English/ Spanish determined by language use)<br><br>(Pfefferbaum et al., 1990, Adams, 1988, Adams, 1989)	3-15.9	PBCL	35	**Convergent validity: Significant**<br>r=not reported, p=not reported in Anglo children	?	Doubtful									
				43	**Convergent validity: Significant**<br>r=not reported, P=not reported in Hispanic children	?	Doubtful									
FPS-R	Brazil (Brazilian Portuguese)<br><br>(Da Silva et al., 2011)	7-17	gender	90	**Divergent validity: Not significant**<br>FPS-R-B r = not reported, p =0.75	-	Doubtful						124	Measurement error:<br>expected to have a high account of measurement error due to it being a single item instrument	?	Inadequate
			FLACC-B	90	**Convergent validity: Significant**<br>FPS-R-B rs=0.74	+	Very good									
	Canada (English)<br><br>(Hicks et al., 2001)	4-12						CAS	45	**Predictive validity: significant**<br>FPS-R r = 0.84	+	Very good				
								VAS	45	**Predictive validity: significant**<br>FPS-R r = 0.92	+	Very good				
	Spain (Catalan)<br><br>(Miro and	7-15	CAS	124	**Predictive validity: Significant**<br>FPS-R-C r=0.87, p<0.001	+	Very good	FAS	124	**Predictive validity: significant**<br>FPS-R-C r = 0.32 p <0.01	-	Very good				

	Huguet, 2004)															
HEDEN	France (French)<br><br>(Marec-Berard et al., 2015)	2-6	DEGR	59	**Convergent validity:**<br>r = 0.5, p = not reported	-	Very good						59	Interrater agreement:<br>r = 0.62	-	Doubtful
				48	**Convergent validity:**<br>r = 0.6, p = not reported	-	Very good						48	Interrater agreement:<br>r = 0.67 (95% CI: 0.48–0.79)	-	Doubtful
MIPS	United States (English)<br><br>(Buchholz et al., 1998)	<1						VAS (nurses)	40	acceptable concordance between MIPS and observer VAS	+	Inadequate	40	Interrater agreement:<br>r = 0.85, p = n.r.	+	Doubtful
MSAS (7-12)	United Kingdom (English)<br><br>(Collins et al., 2002)	7-12						PAIN-VAS	149	**Concurrent validity: Significant**<br>MSAS-PAIN r = 0.76 p<0.01	+	Very good	149	Interrater agreement:<br>for pain subscale, parent and child agreement:<br>weighted kappa = 0.46	-	Very good
OMDQ	Canada (English)<br><br>(Tomlinson et al., 2011)	1-11											59	**Test-retest of pain construct: Significant**<br>Day 1 and 2: **rs** = 0.676 (p <0.0001)<br>Day 14 and 15: **rs** = 0.889 (0.0001)	+	Doubtful
	Canada (English)<br><br>(Manji et al., 2012)	12-17	WHO mucositis	15	**Convergent validity: Significant**<br>OMDQ-Pain rs = 0.90, p<0.0001	+	Adequate						15	**Test-retest: Significant**<br>amount of mouth and throat pain<br>rs = 0.88, P<0.0001	+	Doubtful
			VAS mucositis	15	**Convergent validity: Significant**<br>OMDQ-Pain rs = 0.81, p<0.0001	+	Adequate									
			FACTECS swallow	15	**Convergent validity: Significant**<br>OMDQ-Pain rs = -0.77, p<0.0001	+	Adequate									
			FACTECS eat	15	**Convergent validity: Significant**<br>OMDQ-Pain rs = -0.62, p<0.0001	-	Adequate									
			FACTECS total	15	**Convergent validity: Significant**<br>OMDQ-Pain rs = -0.71, p<0.0001	+	Adequate									
OPS	United States (English)<br><br>(West et al., 1994)	5-13	FPS (parent)	13	**Convergent validity: Significant**<br>τ = 0.26, p =0.10	-	Inadeuqate	FPS (self-report)	13	Concurrent validity<br>τ = 0.37, p =0.02	-	Inadeuqate				
			PCT (parent)	13	**Convergent validity: Significant**<br>τ = 0.22, p =0.16	-	Inadeuqate	PCT (self-report)	13	Concurrent validity<br>τ = 0.27, p =0.09	-	Inadeuqate				
Pain Squad	Canada (English)<br><br>(Stinson et al., 2015)	8-18	generic HRQL	92	Convergent validity:<br>Pain Squad VAS r = (-0.20 to -0.46)	-	Very good									
			disease-specific HRQL	92	Convergent validity:<br>Pain Squad VAS r = (-0.12 to -0.28)	-	Very good									
			pain coping HRQL	92	Convergent validity:<br>Pain Squad VAS r = (0.25-0.29)	-	Very good									
PBCL	United States (English/ Spanish determined by language	3-15.9	Le Baron and Zeltzer, 1984 / Kuttner and LePage,	35	**Convergent validity: Significant**<br>r=not reported, p=not reported in Anglo children	?	Doubtful	self-report PBCL	35	**Concurrent validity: Significant**<br>caregiver PBCL r= 0.04, p <0.01 in Anglo children	-	Very good				
				43	**Convergent validity: Significant**<br>r=not reported, P=not reported in Hispanic children	?	Doubtful	self-report PBCL	43	Concurrent validity: not correlated<br>caregiver PBCL r = 0.14, p <0.01 in Hispanic children	-	Very good				

	use) (Pfefferbaum et al., 1990)		1989 Faces Scale													
			CPI	35	**Convergent validity:** r=0.66, P<0.001 in Anglo children	-	Very good									
			CPI	43	**Convergent validity:** r=0.64, P<0.001 in Hispanic children	-	Very good									
	United States (English) (LeBaron and Zeltzer, 1984)	6-18	Patient self-rating of pain	22	**Convergent validity: significant** r=0.49, 0.53, 0.21, p<0.001, P<0.001, not significant	-	Doubtful						22	**Interrater agreement:** r = 0.64, 0.80, 0.86, p <.01	?	Doubtful
			Observer rating of pain	22	**Convergent validity: significant** r=0.42, 0.64, 0.45, p<0.001	-	Doubtful									
PCT	United States (English) (West et al., 1994)	5-13	WBS (parent)	30	**Convergent validity:** Kendall's = 0.7 (PCT parent) P=0.0001	+	Doubtful	WBS	30	**Concurrent validity: significant** Kendall's=0.67, P=0.0001	-	Doubtful	30	**Interrater agreement:** not significant, value not reported	?	Doubtful
			OPS (nurse)	30	**Convergent validity:** Kendall's = 0.0.27 (PCT patient) P=0.09	-	Doubtful						30	**Intrarater agreement:** Kendall's = 0.23, p=0.16	?	Doubtful
PII	United States (English) (Martin et al., 2015)	6.6-24.1	PII-P	60	**Concurrent validity:** r = 0.62, P<0.0001	-	Very good									
PII-P	United States (English) (Martin et al., 2015)	6.6-24.1						PII (self-report)	60	**Concurrent validity:** r = 0.62, P<0.0001	-	Very good				
PPP	United Kingdom (English) (Hunt et al., 2004)	1-18											140	**Interrater agreement:** **ICC=0.74-0.89**	+	Adequate
	United Kingdom (English) (Hunt et al., 2007)	1-18	Saliva cortisol concentration	29	**Concurrent validity:** rs=0.375-0.451	-	Very good	NRS	29	**Concurrent validity:** r = 0.91	+	Very good	3	**Interrater agreement:** ICC= 0.62 (95%CI: 0.54-0.70) for a single rater; 0.83 (95%CI:0.78-0.88); range: 0.21-0.71, average: 0.45	+	Adequate
														**Intrarater agreement** ICC=0.90 (95%CI: 0.80-0.95, range: 0.21-1.0, average: 0.72)	+	Adequate
	Brazil (Brazilian Portuguese) (Pasin et al., 2013)	1-18												**test-retest:** Adequate stability over time: no significant difference between test and retest (P = 0.271)	-	Doubtful
RPS	Canada (English) (Mahon et al., 2015)	5-10						FPS-R	49	**Concurrent validity:** r = 0.96 (first), 0.97 (second), 0.93 (third) clinic visit κ = 1.0 (first), 0.95 (second), 0.87 (third) clinic visit	+	Very good	49	**Interrater agreement:** Exact agreement between choices made by the participants on both scales: 92% (first), 91% (second), and 87% (third) clinic visit	+	Doubtful
SSPedi	Canada (English)	8-18.7	SSPedi scores of more and	502	**Divergent validity: Significant** Mean difference = 7.8 (95% CI=6.4 to 9.2)	+	Very good						502	**test-retest:** ICC = 0.88 (95% CI=0.82 to 0.92)	+	Adequate

	(Dupuis et al., 2018)		less symptomatic													
			FPS-R	502	**Convergent validity: Significant** Pain items r = 0.52 (95% CI = 0.46 to 0.59), P < .001	-	Very good									
WBS	Lebanon (Arabic) (Badr Zahr et al., 2006)	4-10	OSBD-R (nurses)	45	**Convergent validity: Significant** r = 0.81, p<0.01	+	Very good	DOLLS	45	**Concurrent validity:** r = 0.90, p<0.01	+	Very good				
			FLACC (nurses)	45	**Convergent validity: Significant** r = 0.78, p<0.01	+	Very good									
			OSBD-R (parents)	45	**Convergent validity: Significant** r = 0.73, p<0.01	+	Very good									
			WBS (parents)	45	**Convergent validity: Significant** r = 0.79, p<0.01	+	Very good									
			systolic blood pressure	45	**Convergent validity: Significant** r = 0.59, 0.78, 0.91, p<0.001	+	Very good									
			heart rate	45	**Convergent validity: Significant** r = 0.82, 0.71, 0.85, p<0.01	+	Very good									
			oxygen saturation	45	Convergent validity: no correlation r = not reported, p = not reported	-	Very good									
	United States (English) (Holdsworth et al., 1997)	n.r.	OSBD	336	**Convergent validity: Significant** rs >= 0.807	+	Very good									
	United States (English) (West et al., 1994)	5-13											30	Interrater agreement: significant, value not reported	?	Doubtful
	United States (English) (Wiener et al., 2017)	7-21						Distress Thermometer checklist item: Pain	289	**Concurrent validity: Significant** r = 0.25 and r = 0.28, p<0.001	-	Adequate				

References:

1. Fernandes A, Batalha L, Perdigão A, de Campos C, Nascimento L, Jacob E. Cultural Validation of the Adolescent Pediatric Pain Tool (APPT) in portuguese children with cancer. Revista de Enfermagem Referência. 2015;4(4):99-105.
2. Özalp Gerçeker G, Bilsin E, Binay Ş, Bal Yılmaz H, Jacob E. Cultural adaptation of the Adolescent Pediatric Pain Tool in Turkish children with cancer. European Journal of Oncology Nursing. 2018;34:28-34.
3. Ambuel B, Hamlett KW, Marx CM, Blumer JL. Assessing distress in pediatric intensive care environments: the COMFORT scale. J Pediatr Psychol. 1992;17(1):95-109.
4. van Dijk M, de Boer JB, Koot HM, Tibboel D, Passchier J, Duivenvoorden HJ. The reliability and validity of the COMFORT scale as a postoperative pain instrument in 0 to 3-year-old infants. Pain. 2000;84(2-3):367-77.
5. Pfefferbaum B, Adams J, Aceves J. The influence of culture on pain in Anglo and Hispanic children with cancer. J Am Acad Child Adolesc Psychiatry. 1990;29(4):642-7.
6. Adams J. The assessment of pain related to invasive procedures in Anglo and Hispanic children with cancer. Journal of the Association of Pediatric Oncology Nurses. 1988;5(1-2):31.
7. Adams JN. A methodologic study of pain assessment in Anglo and Hispanic children with cancer 1989.
8. Gauvain-Piquard A, Rodary C, Rezvani A, Lemerle J. Pain in children aged 2-6 years: a new observational rating scale elaborated in a pediatric oncology unit--preliminary report. Pain. 1987;31(2):177-88.
9. Gauvain-Piquard A, Rodary C, Rezvani A, Serbouti S. The development of the DEGR(R): A scale to assess pain in young children with cancer. European Journal of Pain. 1999;3(2):165-76.
10. Marec-Berard P, Gomez F, Combet S, Thibault P, Le Moine P, Bergeron C. HEDEN Pain Scale: A Shortened Behavioral Scale for Assessment of Prolonged Cancer or Postsurgical Pain in Children Aged 2 to 6 Years. Pediatric Hematology and Oncology. 2015;32(5):291-303.
11. Da Silva FC, Santos Thuler LC, de Leon-Casasola OA. Validity and reliability of two pain assessment tools in Brazilian children and adolescents. Journal of Clinical Nursing. 2011;20(13-14):1842-8.
12. Miro J, Huguet A. Evaluation of reliability, validity, and preference for a pediatric pain intensity scale: the Catalan version of the faces pain scale--revised. Pain. 2004;111(1-2):59-64.
13. Collins JJ, Devine TD, Dick GS, Johnson EA, Kilham HA, Pinkerton CR, et al. The measurement of symptoms in young children with cancer: The validation of the memorial symptom assessment scale in children aged 7-12. Journal of Pain and Symptom Management. 2002;23(1):10-6.
14. Stinson J, Jibb LA, Nathan PC, Maloney AM, Dupuis L, Gerstle JT, et al. Construct validity and reliability of a multidimensional electronic pain diary for school-aged children and adolescents with cancer. Pediatric Blood and Cancer. 2015;62 (Supplement 4):S194.
15. Martin S, Nelson Schmitt S, Wolters PL, Abel B, Toledo-Tamula MA, Baldwin A, et al. Development and Validation of the English Pain Interference Index and Pain Interference Index-Parent Report. Pain Medicine (United States). 2015;16(2):367-73.
16. Pasin S, Avila F, De Cavata T, Hunt A, Heldt E. Cross-cultural translation and adaptation to Brazilian Portuguese of the paediatric pain profile in children with severe cerebral palsy. Journal of Pain and Symptom Management. 2013;45(1):120-8.
17. Hunt A, Wisbeach A, Seers K, Goldman A, Crichton N, Perry L, et al. Development of the Paediatric Pain Profile: Role of Video Analysis and Saliva Cortisol in Validating a Tool to Assess Pain in Children with Severe Neurological Disability. Journal of Pain and Symptom Management. 2007;33(3):276-89.

18. Hunt A, Goldman A, Seers K, Crichton N, Mastroyannopoulou K, Moffat V, et al. Clinical validation of the paediatric pain profile. Dev Med Child Neurol. 2004;46(1):9-18.
19. Mahon P, Holsti L, Siden H, Strahlendorf C, Turnham L, Giaschi D. Using Colors to Assess Pain in Toddlers: Validation of "The Rainbow Pain Scale"—A Proof-of-Principle Study. Journal of Pediatric Oncology Nursing. 2015;32(1):40-6.
20. Dupuis LL, Johnston DL, Baggott C, Hyslop S, Tomlinson D, Gibson P, et al. Validation of the symptom screening in pediatrics tool in children receiving cancer treatments. Journal of the National Cancer Institute. 2018;110(6):661-8.
21. Van Dijk M, De Boer JB, Koot HM, Duivenvoorden HJ, Passchier J, Bouwmeester N, et al. The association between physiological and behavioral pain measures in 0- to 3-year-old Infants after major surgery. Journal of Pain and Symptom Management. 2001;22(1):600-9.
22. Badr Zahr LK, Puzantian H, Abboud M, Abdallah A, Shahine R. Assessing procedural pain in children with cancer in Beirut, Lebanon. Journal of Pediatric Oncology Nursing. 2006;23(6):311-20.
23. Merkel SI, Voepel-Lewis T, Shayevitz JR, Malviya S. The FLACC: a behavioral scale for scoring postoperative pain in young children. Pediatr Nurs. 1997;23(3):293-7.
24. Manworren RCB, Hynan LS. Practice applications of research. Clinical validation of FLACC: preverbal patient pain scale. Pediatric Nursing. 2003;29(2):140-6.
25. Hicks CL, von Baeyer CL, Spafford PA, van Korlaar I, Goodenough B. The Faces Pain Scale-Revised: toward a common metric in pediatric pain measurement. Pain. 2001;93(2):173-83.
26. Buchholz M, Karl HW, Pomietto M, Lynn A. Pain scores in infants: a modified infant pain scale versus visual analogue. Journal of Pain & Symptom Management. 1998;15(2):117-24.
27. Tomlinson D, Ethier MC, Judd P, Doyle J, Gassas A, Naqvi A, et al. Reliability and construct validity of the oral mucositis daily questionnaire in children with cancer. European Journal of Cancer. 2011;47(3):383-8.
28. Manji A, Tomlinson D, Ethier MC, Gassas A, Maloney AM, Sung L. Psychometric properties of the Oral Mucositis Daily Questionnaire for child self-report and importance of mucositis in children treated with chemotherapy. Supportive Care in Cancer. 2012;20(6):1251-8.
29. West N, Oakes L, Hinds PS, Sanders L, Holden R, Williams S, et al. Measuring pain in pediatric oncology ICU patients... including commentary by Wong DL. Journal of Pediatric Oncology Nursing. 1994;11(2):64-70.
30. LeBaron S, Zeltzer L. Assessment of acute pain and anxiety in children and adolescents by self-reports, observer reports, and a behavior checklist. J Consult Clin Psychol. 1984;52(5):729-38.
31. Holdsworth MT, Raisch DW, Winter SS, Chavez CM, Leasure MM, Duncan MH. Differences among raters evaluating the success of EMLA cream in alleviating procedure-related pain in children with cancer. Pharmacotherapy:The Journal of Human Pharmacology & Drug Therapy. 1997;17(5):1017-22.
32. Wiener L, Battles H, Zadeh S, Widemann BC, Pao M. Validity, specificity, feasibility and acceptability of a brief pediatric distress thermometer in outpatient clinics. Psycho-Oncology. 2017;26(4):461-8.
